# Gas Stoves in Bed-Rooms

**Published:** 1899-02-18

**Authors:** 


### EDITOR'S LETTER-BOX.

[Our Correspondents are reminded that prolixity is a great bar to publication, and that brevity of style and conciseness of statement greatly facilitate early insertion.]

### GAS STOVES IN BED-ROOMS.

"An Architect" writes: Are gas-stoves dangerous or undesirable in a sick room? A doctor of my acquaintance always says, "Take away that horrid thing," or "take her (the patient) into a room with a decent fire," when he is called in to attend a case and finds one of Fletcher's very best contrivances in use. I need not point out the great convenience of a well constructed gas-stove; the certainty with which the temperature can be regulated, the absence of all noise from putting on coals, of smoke, and of all attention beyond turning a tap, and the great saving of labour in carrying coals where the household is not over-blessed with servants. A gas-stove with an unsuitable flue, so that the products of combustion enter the room, or one which, from ignorance or ill-construction, is always "lighted back," is, of course, no proper substitute for a coal fire, and should at once be removed or amended; but I should be grateful if I could obtain an authoritative answer to the question at the commencement of this letter, assuming that the stove is properly constructed, is properly lighted and has a good up-draught at all times.

*** We presume that "An Architect" refers to open asbestos or "cobble" fires with "atmospheric" or Bunsen burners. The question is an important and interesting one. There is no doubt that gas stoves are neither "dangerous nor undesirable" when the conditions mentioned by "An Architect" co-exist, namely when "the stove is properly constructed, is properly lighted, and has a good up-draught at all times." Much, however, hinges on the term, "properly constructed," which, of course, includes "properly placed." The products of combustion with gas and coal are practically the same, with the exception of the soot and tarry compounds which are produced by coal, and if the provision made for their removal is the same, the result is the same. Unfortunately, gas is expensive, and the chief, perhaps the only way, in which its use can be made economical, is by so arranging the stove that a far smaller proportion of the heat shall go up the chimney than is the case with coal, and this is the real cause of much of the evil which undoubtedly does often attend the use of gas fires. Everyone admits the wastefulness of the open fire. This depends partly on the fact that a very large proportion of the heat goes up the chimney, and partly on the fact that in order to carry away all the smoke (which, being visible, would soon show if it were not carried away), the stove is so constructed that there is a constant up-draught from the whole of the front of the fireplace. By this means a great quantity of air is carried up the chimney which never goes through the fire at all. But this apparent wastefulness is not all waste, because while some of the heat which goes up the chimney serves to improve the draught, the great quantity of unburnt air which also goes up it, although cooling the room by drawing fresh supplies from outside, does at least increase the ventilation. But when the maker of a gas stove for the sake of economy lessens the proportion of the heat and also the amount of air which goes up the chimney, although he makes a very good stove, he does diminish the ventilation of the room. It must be remembered that the virtues of the open fire, and the very things for which it is loved, depend upon its being "wasteful," as the saying is, and that if we want those same virtues in a gas fire it also must be constructed on the same "wasteful" plan—must be put under the chimney and have a free access of air—and that gas being more expensive than coal the loss will be all the greater. The advantages of gas fires, however, are so great that in many cases we may willingly put up with a certain amount of "waste" of gas for their sake. Moreover, it must be said that much depends upon the chimney. With a chimney that draws of itself and draws always a much more economical gas stove may be employed than would be safe with a fickle chimney which requires to be kept warm to make it act.

